# Corrosion Behavior of As-Cast and Heat-Treated Al–Co Alloys in 3.5 wt% NaCl

**DOI:** 10.3390/ma17030655

**Published:** 2024-01-29

**Authors:** Athanasios K. Sfikas, Angeliki G. Lekatou, Sevasti Emmanouilidou, Kyriaki Tsirka

**Affiliations:** 1 Laboratory of Applied Metallurgy, Department of Materials Science and Engineering, University of Ioannina, 45110 Ioannina, Greece; thanasfi@gmail.com (A.K.S.); sevina.emma@gmail.com (S.E.); 2 Institute of Materials Science and Computing, University Research Centre of Ioannina (URCI), 45110 Ioannina, Greece; 3 Composites and Smart Materials Laboratory, Department of Materials Science and Engineering, University of Ioannina, 45110 Ioannina, Greece; ktsirka@uoi.gr

**Keywords:** Al–Co alloys, Al_9_Co_2_, Al_13_Co_4_, stir casting, heat treatment, X-ray diffraction, microstructure, reverse polarization, chronoamperometry, pitting

## Abstract

The present work evaluates the effect of Co content on the microstructure and corrosion performance of Al–Co alloys of various compositions (2–32 wt% Co), fabricated by flux-assisted stir casting. A preliminary investigation on the effect of heat treatment (600 °C, up to 72 h) on the microstructure and corrosion behavior of Al–20 wt% Co and Al–32 wt% Co was also conducted. The Al– (2–10) wt% Co alloys were composed of acicular Al_9_Co_2_ particles uniformly dispersed in an Al matrix. The Al–20 wt% Co and Al–32 wt% Co alloys additionally contained Al_13_Co_4_ blades enveloped in Al_9_Co_2_ wedges. Heat treatment of Al–20 wt% Co and Al–32 wt% Co led to a significant reduction in the volume fraction of Al_13_Co_4_ and a decrease in hardness. Al–Co alloys with high Co content (10–32 wt% Co) exhibited greater resistance to localized corrosion in 3.5 wt% NaCl, but lower resistance to general corrosion compared to the (0–5 wt% Co) alloys. Heat treatment led to a slight increase in the corrosion resistance of the Al–Co alloys. The microstructure of the produced alloys was analyzed and correlated with the corrosion performance. Finally, corrosion mechanisms were formulated.

## 1. Introduction

Al–Co alloys have attracted a lot of attention in the last few years due to their good mechanical [1], corrosion [2], oxidation [3], and wear properties [4]. Aluminum alloys in this unusual category have been fabricated using different techniques, including vacuum arc melting (VAM) [5], powder metallurgy [6], casting [7], additive manufacturing [8], melt spinning [9], and thermal explosion [10,11,12]. Furthermore, Al composites reinforced by Co-aluminide particles have successfully been produced with the employment of powder metallurgy techniques involving the in situ formation of intermetallic reinforcement due to a reaction between Al and Co powder during sintering [13,14], or the ex situ addition of the intermetallic reinforcement (Al_9_Co_2_ produced by VAM) into the Al powder followed by sintering [15].

A wide range of Al–Co compositions, from 2 wt% Co to 47.2 wt% Co, were produced using vacuum arc melting, resulting in diverse microstructures [4,16,17]. Lower Co compositions (2–20 wt% Co) consisted of (Al) and Al_9_Co_2_ [4], while higher Co compositions (40.8–47.2 wt% Co) were entirely intermetallic, including aluminides like Al_13_Co_4_, Z-Al_3_Co, and Al_5_Co_2_ [16,17]. VAM Al–Co alloys with Co in the range of 2–32 wt% Co (Al-Al_9_Co_2_) exhibited directional microstructures, transitioning from eutectic to supereutectic with increasing Co content [4,18]. Notably, relatively high amounts of Co were found dissolved in the Al matrix (ranging from 0.5 wt% in Al–2 wt% Co to 5.5 wt% in Al–20 wt% Co) [4,19], despite the negligible Co solubility in Al under equilibrium conditions [20]. This was attributed to the high solidification rates involved in the fabrication process [19].

High transition metal (TM) content in Al-based surface films is generally considered to improve the pitting resistance of binary Al–TM alloys in chloride solutions [21]. While limited studies have focused on Al–Co alloy corrosion, existing research on VAM processed Al–Co alloys, suggests good corrosion resistance [18,19,22]. In further detail, VAM Al–Co alloys exhibited low susceptibility to localized corrosion in 3.5 wt% NaCl; localized corrosion evolved from (Al) pitting to crevice corrosion, along the Al–aluminide interface [19]. In 1M H_2_SO_4_, the increasing Co content improved the passivation performance [22]. Al–Co intermetallics with high Co contents displayed improved corrosion performance in aqueous NaCl [16,17]. Recently, Palcut et al. reported that Al–2.2 wt% Co exhibited lower corrosion resistance than Al and Al–6.3 wt% Co in aqueous NaOH, attributed to the preferential attack on the Al/Al_9_Co_2_ eutectic [23].

The impact of preparation methods on the microstructure and surface degradation performance of Al–Co alloys has been the focus of previous efforts, dealing with two alloy compositions (7 and 32 wt% Co) [6,18]. Vacuum arc melting demonstrated superior corrosion and sliding wear performance compared to methods with lower solidification rates or solid-state diffusion. This was attributed to lower porosity, finer microstructures, and increased Co solubility in the Al matrix in VAM alloys [6,18]. Nevertheless, these two efforts showed that conventionally cast Al–7 wt% Co and Al–32 wt% Co still exhibited fairly similar localized corrosion resistance, whilst demonstrating slightly inferior electrochemical values in a saline environment, compared to their VAM-processed counterparts.

Simple measures in conventional casting, such as: (i) adding wetting agents (usually fluoride salts) to enhance the close contact between the molten metal and second phase particles, achieved by reducing the surface tension of the liquid phase and dissolving surface oxides, and (ii) employing mechanical agitation to limit segregation, ensure temperature uniformity and promote the preferred wetting behavior with a contact angle below 90°, can improve the wettability, while maintaining reasonable fabrication costs [24,25,26,27].

Taking all the above into consideration, this effort examines the microstructure and corrosion performance of Al–Co alloys produced through a low cost and flexible technique like stir casting, suitable for conventional foundries without the need for equipment changes. Different Co contents, spanning from 2 wt% Co to 32 wt% Co, were selected to assess the impact of alloy composition on the microstructure and corrosion properties. Additionally, this study aims to evaluate the influence of heat treatment (HT) on the microstructure and corrosion performance of the Al–Co alloys with the highest Co content and the most complex microstructure. To the authors’ knowledge, this effort represents the first comprehensive investigation of a broad range of Al–Co alloy compositions fabricated by stir casting and the assessment of their microstructure and corrosion behavior in both as-cast and heat-treated configurations.

## 2. Materials and Methods

The Al–Co alloys (2, 5, 10, 20, 32 wt% Co), hereafter denoted as Al–2Co, Al–5Co, Al–10Co, Al–20Co and Al–32Co, were produced using Al1050 in small pieces (~2 × 2 cm), cut from a 3 mm thick sheet, Co powder (99.5% purity, grain size < 37 μm), and KBF_4_ fluxing agent. The latter was added to promote the contact between Co and the Al melt, by reacting with the Al melt and forming a surface layer of slag that dissolved surface oxides [24,25]. The flux-assisted casting involved melting Al1050 at 850 °C, adding a mixture of Co powder and KBF_4_ under continuous stirring (3220 rpm, ~20 s), removing the slag using a ladle, and casting into cylindrical graphite molds (15 mm diameter, 150 mm height). Al–20Co and Al–32Co samples underwent heat treatment at 600 °C for 24 h, 48 h, and 72 h. Heat treatment was preliminarily applied only to the highest Co compositions, as they were the ones of greatest interest due to the high hardness, although at the likely expense of ductility. 

The cylindrical bars obtained were cut into smaller pieces (15 mm diameter, 10 mm height) using a diamond saw. Specimens were embedded in phenolic resin and underwent standard metallographic procedures. X-ray diffractograms were acquired using a Bruker D-8 Advance diffractometer (CuKa radiation, step size: 0.02^o^, step time: 2 s/step, Bruker, MA, USA). Microstructural analysis utilized a Leica DM-400 optical microscope (OM, Leica Microsystems, Wetzlar, Germany), Jeol JSM 5600 and Jeol 6510 LV scanning electron microscopes (SEM in backscattered electron (BEC) and secondary electron (SE) modes, JEOL, Tokyo, Japan), equipped with an Oxford Instruments EDX detector (Oxford Instruments, High Wycombe, UK). The hardness (HB2.5 under 613 N load) of the alloys was measured using polished cross-sections of 3 replicate samples (mean of 5 measurements per sample, Inovatest IN-700M, Innovatest Europe BV, Maastricht, The Netherlands).

The corrosion performance of the Al–Co alloys (as cast and heat treated) was assessed as follows: samples were ground to 1000 grit by SiC papers (attaining a surface roughness of Ra ≤ 0.2 μm), and encapsulated in PTFE, exposing a ~1 cm^2^ free surface. Potentiodynamic polarization testing was conducted in 3.5 wt% NaCl, at room temperature (RT), using the ACM Gill AC galvanostat/potentiostat (ACM Instruments, Cumbria, UK). The standard three-electrode cell used included the working electrode (Al–Co specimen), a reference electrode (Ag/AgCl/3.5 M KCl), and an auxiliary electrode (platinum gauge). After a 4 h open-circuit immersion, the potentiodynamic polarization started at a scanning rate of 10 mV/min. 

The corrosion current density (i_corr_) values were calculated using Tafel extrapolation through linear regression applied to the potential against the logarithm of the current density data. Accuracy was pursued by complying with specific restrictions [18,19]: (i) the linear fit started at potentials 100 mV away from the corrosion potential to diminish any interinfluence between anodic and cathodic reactions and, consequently, any deviation from the Tafel-like behavior at low current densities; (ii) regression spanned at least one order of magnitude of the current density, so as to minimize any aberration caused by concentration polarization and external effects like surface roughening; as such, Tafel extrapolation was performed only on the cathodic portion, since it yielded an extended and better delineated Tafel region; (iii) a low scan rate was employed (10 mV/min) to minimize the capacitive effects at the electrode–electrolyte interface; (iv) the linear fit was deemed satisfactory only when the regression coefficient exceeded 0.98; (v) only one reduction reaction should occur within the linear fit range (the oxygen reduction reaction prevails in naturally aerated 3.5 wt% NaCl).

Cyclic potentiodynamic polarization was applied to assess the resistance to localized corrosion. The technique relies on the occurrence of localized corrosion when the current density in the anodic curve of the reverse scan surpasses that of the forward scan at the same anodic potential, because of enhanced corrosion conditions within developing pits. This is represented by the formation of a clockwise hysteresis loop [28]. Four replicate polarization runs were conducted for each composition. Chronoamperometry testing was conducted, aiming to validate the findings of the potentiodynamic polarization testing and further investigate the corrosion processes in relation to Co content. “Current density against immersion time” curves were recorded during 2 h immersion in aerated 3.5 wt% NaCl at RT, at potentials in stages identified in the earlier potentiodynamic anodic scans.

## 3. Results and Discussion

### 3.1. Microstructure of the As-Cast Alloys

**Figure 1:**
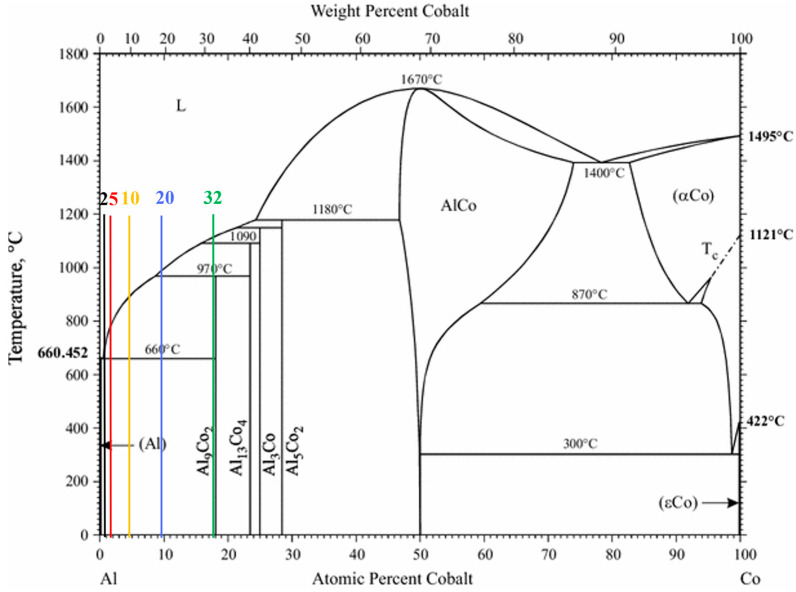
Al–Co equilibrium phase diagram [29] and the studied compositions indicated by vertical lines of different colors.

The compositional range of 2–32 wt% Co corresponds to the hypereutectic regime of the Al–Al_9_Co_2_ phase diagram (Figure 1), so the expected microconstituents would be primary Al_9_Co_2_ and eutectic Al/Al_9_Co_2_ [29].

**Figure 2:**
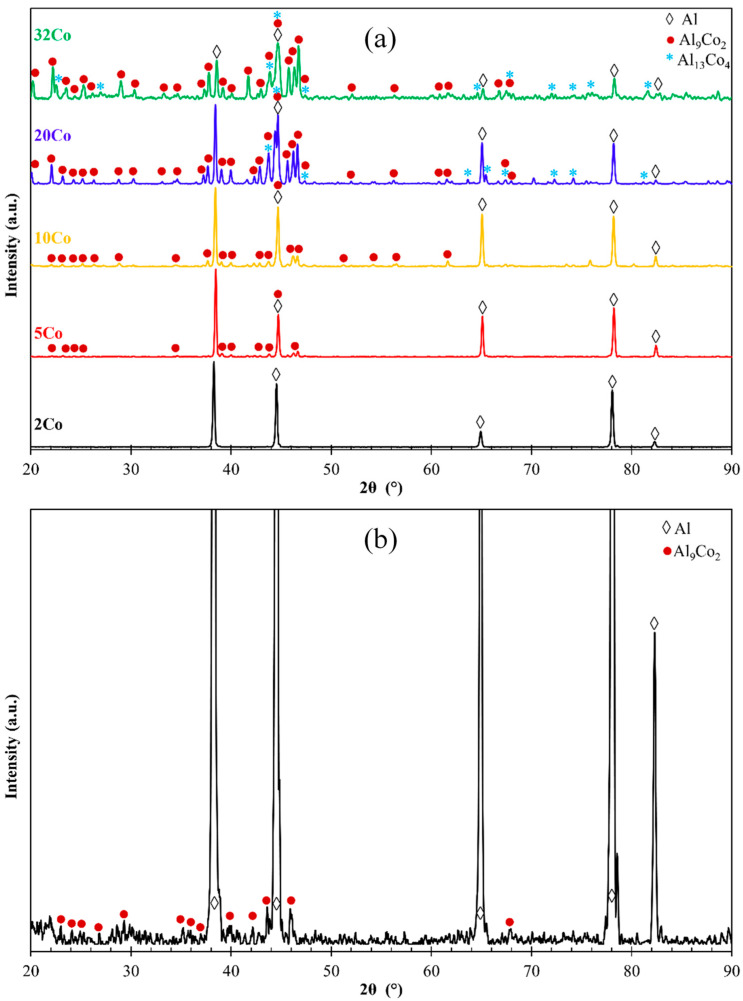
(**a**) XRD patterns of the as-cast Al–Co alloys (powder diffraction files: αAl: 4-0787, Al_9_Co_2_: 3-0007 and 6-0699, Al_13_Co_4_: 44-1304 and 50-0694), (**b**) magnified XRD pattern of Al–2 wt% Co for Al_9_Co_2_ assignments.

Figure 2 displays the XRD patterns of the Al–Co alloys. The Al– (2-10)Co alloys consist of Al and Al_9_Co_2_. Al_9_Co_2_ peaks are evident in the magnified XRD pattern of Al–2Co (Figure 2b). The intensity of the Al_9_Co_2_ peaks increases with higher Co content, indicating an increasing volume fraction of the intermetallic compound (IC). Al–20Co and Al–32Co exhibit peaks corresponding to Al, Al_9_Co_2_, and Al_13_Co_4_, with noticeable intensity in the Al_13_Co_4_ peaks. Despite the expected equilibrium phases being Al and Al_9_Co_2_, as per the Al–Co phase diagram, Al_13_Co_4_ is also detected at 20 and 32 wt% Co. This discrepancy will be discussed further in the context of the microscopy observations.

**Figure 3:**
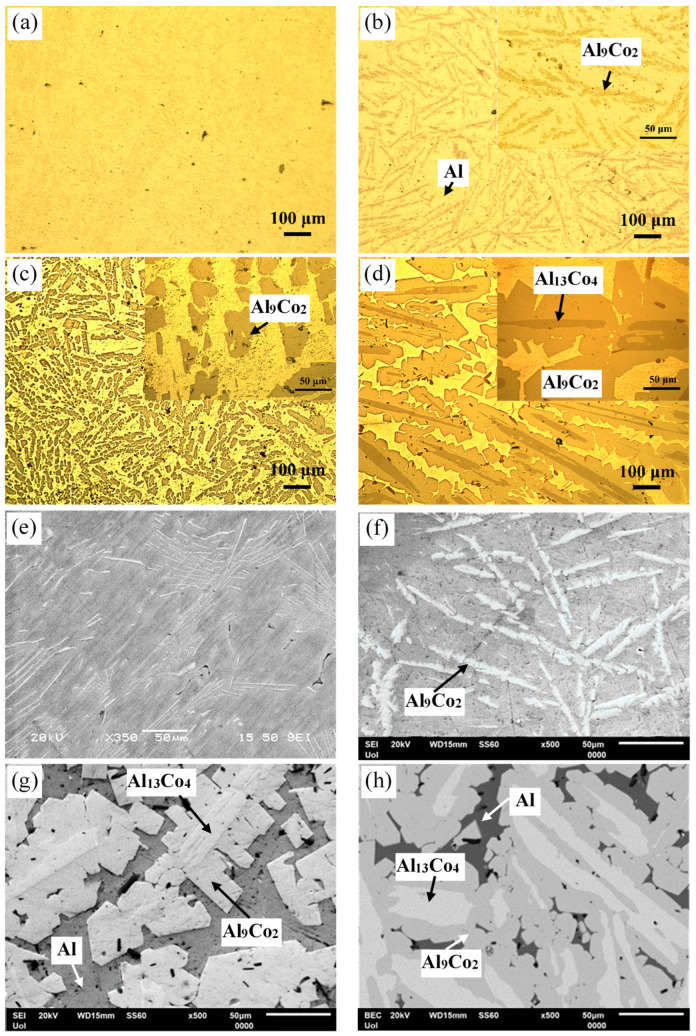
Microstructure of as-cast Al–Co alloys: OM (**a**) Al–2Co, (**b**) Al–5Co, (**c**) Al–10Co, (**d**) Al–20Co; SEM (**e**) Al–2Co, (**f**) Al–5Co, (**g**) Al–20Co, (**h**) Al–32Co.

Figure 3 manifests that the as-cast Al– (2-10) wt% Co alloys exhibit a microstructural pattern of acicular hard phases, uniformly dispersed in a soft Al matrix. The thickness of the dispersed phases increases with higher Co content, ranging from fine needles in Al–2Co (Figure 3a) to coarse, longish wedges in Al–10Co (Figure 3c). Quantitative EDX analysis reveals the composition of Al_9_Co_2_. As the Co content increases, the content of primary Al_9_Co_2_ increases and this reflects on the increase in the coarseness of the Al_9_Co_2_ needles. In the instance of Al–5Co, the intermetallic blades have grown in a brick-like pattern (Figure 3f), attributed to a reduction in the required undercooling for the uninterrupted growth of crystals. Consequently, as the Al_9_Co_2_ solidification progresses, there is a concurrent decrease in the cooling rate. This dynamic leads to a decrease in the diffusivity of Co atoms in the liquid, and the liquid in front of the Al_9_Co_2_ “brick”, which is ready to solidify, is being depleted of Co [30,31].

In Al–20Co, the Al_9_Co_2_ wedges often envelope blades, which have evolved to bulky blocks in the case of Al–32Co (Figure 3d,g and h, respectively). Quantitative EDX analysis revealed that these core phases have the composition of Al_13_Co_4_, in agreement with the XRD results in Figure 2. The non-expected presence of Al_13_Co_4_ can be attributed to local segregations of Co upon the addition of the powder mixture of Co and KBF_4_ in Al, which has led to the immediate solidification of Al_13_Co_4_ through the spontaneous reaction of liquid Al and Co. It should also be noted that Al_13_Co_4_ has a more negative enthalpy of formation compared to Al_9_Co_2_ and, hence, a greater thermodynamic tendency of formation [32]. The concentric morphology of the intermetallic components in high Co alloys can be explained as follows [18]: The Co-depleted liquid surrounding Al_13_Co_4_ lies in the (liquid + Al_9_Co_2_) regime of the Al–Co phase diagram. As such, upon cooling from 850 to 657 °C, primary Al_9_Co_2_ solidifies around Al_13_Co_4_. At 657 °C, the remaining liquid undergoes the eutectic l → Al + Al_9_Co_2_ reaction, forming Al zones that penetrate Al_9_Co_2_ like pins. This results in an intermetallic structure with a central “trunk” and thick, short branches (Figure 3d,g) supported by the penetrating Al. This “anchorage” arrangement is also observed in the Al–32Co alloy (Figure 3h). The latter composition exhibits a similar phase topology, suggesting a similar solidification sequence to the Al–20Co alloy. Al–32Co exhibits an almost entirely intermetallic structure, comprising Al_9_Co_2_ wedges engulfing Al_13_Co_4_ lumps and thin Al stringers (Figure 3h). 

Figure 3g,h reveals increased porosity at or near the Al_13_Co_4_/Al_9_Co_2_ interface due to differing densities of the two brittle phases. Porosity is also observed at stress concentration points, like narrow interspaces between adjacent dendrite arms. 

Overall, the microstructural study has revealed several microstructural features that may induce various types of corrosion cells, such as composition cells (Al/Al_9_Co_2_ and Al_9_Co_2_/Al_13_Co_4_ boundaries), stress cells (intermetallic blade tips), and differential aeration cells (Al narrow zones at high Co contents, Al/Al_9_Co_2_ interfaces, and Al pins at the periphery of Al_9_Co_2_).

### 3.2. Microstructure of the Heat-Treated Alloys

**Figure 4:**
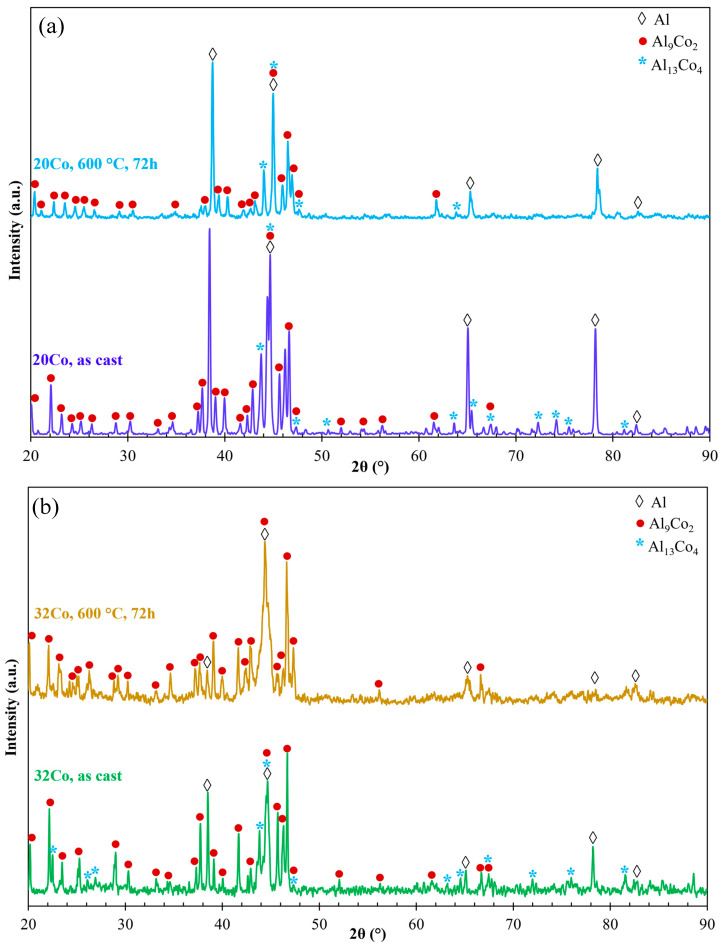
The XRD patterns of (**a**) Al–20Co and (**b**) Al–32Co in the as-cast state and after heat treatment at 600 °C for 72 h (αAl: 4-0787, Al_9_Co_2_: 3-0007 και 6-0699, Al_13_Co_4_: 44-1304 και 50-0694).

Figure 4 depicts the XRD patterns of as-cast and HT Al–20Co and Al–32Co. In HT Al–20, the Al_13_Co_4_ peaks present reduced intensities compared to as-cast Al–20Co, with some peaks even absent. Al_13_Co_4_ peaks are not even detected in HT Al–32Co. Two out of four Al-assigned peaks in the HT alloys appear noticeably reduced, as compared to the as-cast alloys, raising the possibility of a reduction in the Al phase due to HT (2θ = 65.1° and 78.2° in Al–20Co and 2θ = 38.5° and 78.2° in Al–32Co). Therefore, the XRD patterns of Al–20Co and Al–32Co suggest that heat treatment has led to decreased amounts of Al_13_Co_4_ and possibly Al.

**Figure 5:**
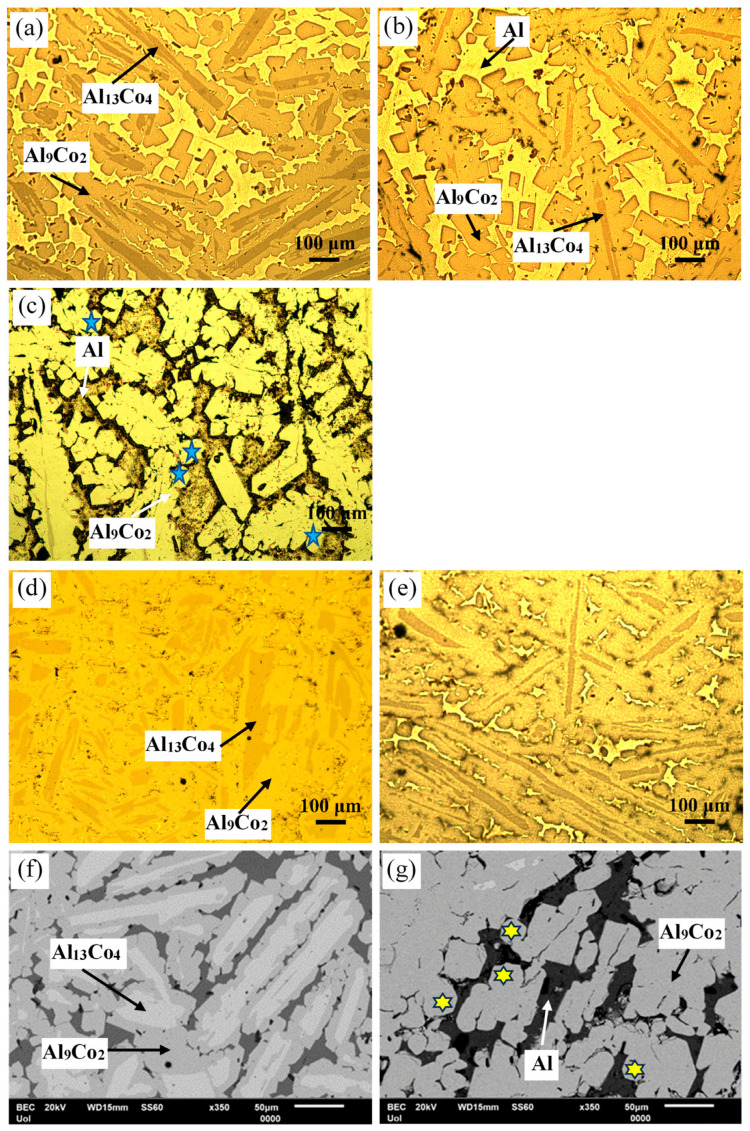
Microstructure of Al–20Co: (**a**) as-cast, (**b**) HT at 600 °C for 24 h, (**c**) HT at 600 °C for 72 h. Microstructure of Al–32Co: (**d**) as-cast, (**e**) HT at 600 °C for 24 h, (**f**) as-cast, (**g**) HT at 600 °C for 72 h (**a**–**e**: OM, **f**,**g**: SEM/BEC). Blue stars in (**c**) and yellow stars in (**g**) point at coarse rounded IC particles.

Figure 5 presents the microstructures of Al–20Co and Al–32Co, as-cast and after HT at 600 °C. For both compositions, the main microstructural effect of heat treatment is a notable reduction in the Al_13_Co_4_ phase with HT time. This indicates a transformation of Al_13_Co_4_ to Al_9_Co_2_ during HT, previously observed in sintering at 600 °C of Al–Co powders [13] and Al powder-Al_9_Co_2_ particles [15]. In fact, the extensive presence of coarse rounded particles in Figure 5c,g (highlighted by blue stars and yellow stars, respectively) raises the possibility of solid-state diffusion processes (sintering and/or annealing) during HT. The porosity in Al_9_Co_2_, seen in Figure 5c,g, enhances the possibility that Al_13_Co_4_ transformed into Al_9_Co_2_ by diffusion of Al through pores. In the as-cast alloys, blades of Al_13_Co_4_ were trapped within Al_9_Co_2_, creating a structure with high residual stresses. Moreover, various Al_13_Co_4_ allotropes are thermodynamically unstable towards Al_9_Co_2_ and Al_5_Co_2_ [33]. Annealing facilitated stress relaxation and transformation of unstable Al_13_Co_4_ to Al_9_Co_2_ through Al uptake.

**Figure 6:**
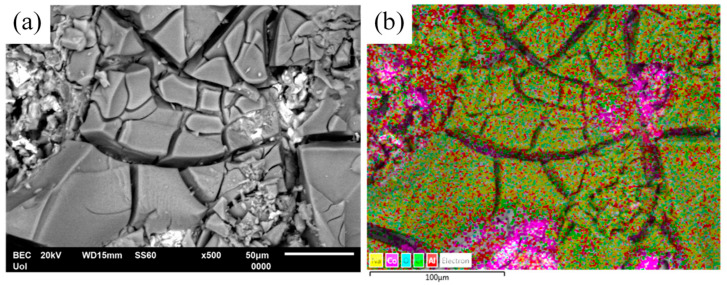
(**a**) SEM/BEC micrograph of Al–32Co heat treated at 600 °C for 72 h, and (**b**) corresponding elemental mapping (yellow: Na; magenta: Co; turquoise: O; light green: Cl; Al: red) showing pockets of Co particles. The specimen has been subjected to cyclic polarization in aqueous NaCl.

This porosity can be attributed to the Kirkendall effect, observed when the diffusion rates of the counter-diffusing species differ [34]. Namely, due to the faster diffusion of Al compared to Co, not every site will be filled by counter-diffusing Co atoms. This creates a vacancy flow opposite to Al diffusion to offset the imbalance in the fluxes of Al and Co. In the absence of sufficient plastic relaxation during this counter-diffusion process, vacancies may combine to form pores or voids within the reaction layer [6]. A prerequisite for this effect is the existence of Co free atoms. Indeed, Figure 6 reveals the presence of Co particles in HT Al–32Co. The diffusion of Al atoms towards free Co atoms has led to the formation of the aluminide that is richest in Al (i.e., Al_9_Co_2_).

To conclude, two likely mechanisms responsible for the elimination of Al_13_Co_4_ and the corresponding increase in Al_9_Co_2_ through heat treatment are proposed: (a) annealing facilitates diffusion of Al into Al_13_Co_4_ through porosity due to the Kirkendall effect and through microcracks due to the inherent brittleness of the intermetallic blades, ultimately leading to the transformation of Al_13_Co_4_ to Al_9_Co_2_; and (b) free Al and Co atoms are combined to form Al_9_Co_2_. These considerations are supported by hardness measurements, with Table 1 showing that the hardness increases with higher Co content, owing to the increased intermetallic compounds. The marked hardness rise in Al–32Co is justified by the significant increase in the harder Al_13_Co_4_ [18]. Heat treatment has reduced the hardness by decreasing Al_13_Co_4_, coupled with the annealing process. The reduction in the hardness with the HT time is more pronounced in Al–32Co compared to Al–20Co, since the elimination of Al_13_Co_4_ is much more extensive. Low Co alloys show a much subtler reduction in hardness compared to high Co alloys, primarily associated with stress relief processes.

### 3.3. Electrochemical Behavior of the As-Cast Alloys

**Figure 7:**
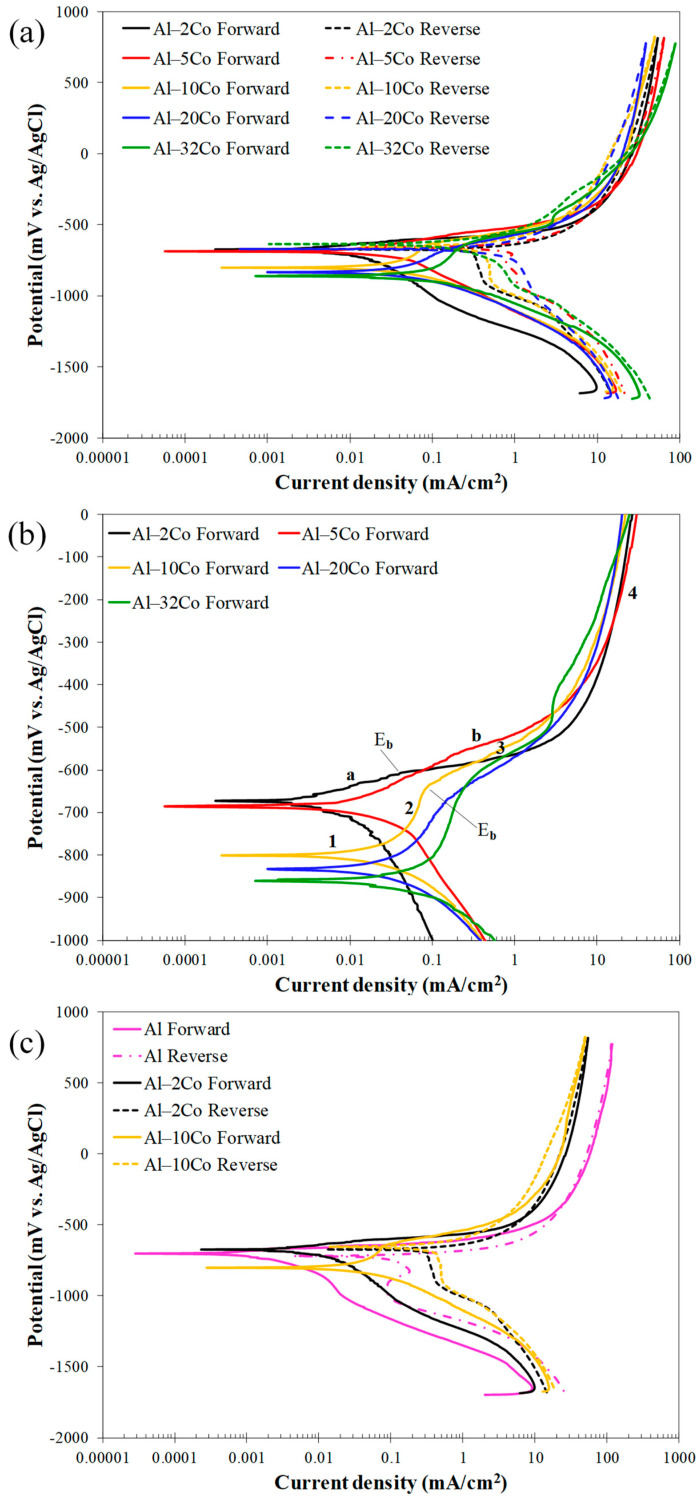
Voltammograms of Al–Co alloys in 3.5 wt% NaCl (RT): (**a**) cyclic polarization, (**b**) forward polarization; letters “a”, ”b” and numbers 1–4 in (**b**) address distinct stages during anodic polarization of alloys Al– (2-5)Co and Al– (10–32)Co, respectively, (**c**) cyclic polarization of stir cast Al1050, Al–2Co, and Al–10Co.

The voltammograms for the stir-cast Al–Co alloys are presented in Figure 7, while the electrochemical values are given in Table 2. 

**Low Co compositions:** Al–2Co and Al–5Co exhibit similar polarization behaviors. In more detail, the anodic portion of the curves can be divided into two stages: an initial stage, where the current increases fast (for more than four orders of magnitude) with a small increase in potential, and a second stage, where the current density values tend to stabilize, at very high values though. A more careful observation of a closer range of anodic potentials, in Figure 7b, reveals that the first stage is actually divided into two sub-stages: stage “a” and stage “b”. During stage b (above E_b_), the current density increase is much faster compared to stage a. Also, the current density increase, during stage b, for Al–2Co is clearly faster than that for Al–5Co.

At high anodic potentials, where the current density tends to stabilize, the current density values of reverse polarization correspond to lower values, as compared to the forward polarization values for the same potentials forming a counter-clockwise hysteresis loop. Nonetheless, at lower potentials, a distinct clockwise hysteresis loop is formed. Considering the persistent increase in the current density in stage a and, especially, stage b, the formation of a clockwise hysteresis loop at lower anodic potentials and the E_b_ values being just a few decades of mV higher than E_corr_ (Table 2), it is implied that Al–2Co and Al–5Co exhibit low resistance to localized forms of corrosion. The similarity in the shape of the forward anodic curves of Al–2Co and Al in Figure 7c, whilst fairly different compared to that of Al–10Co, suggest that the anodic polarization behavior of low Co alloys is governed by the high content of the Al matrix. Therefore, the low resistance of low Co alloys to localized corrosion can be attributed to the low resistance of Al to chloride solutions [19]. The current stabilization at high anodic potentials and high current density values, along with the respective counter-clockwise hysteresis loops, suggest that at high potentials, deposition of unstable products at sites of localized corrosion has occurred. 

**High Co compositions:** On the other hand, Al– (10–32)Co alloys show different polarization behavior, especially regarding the presence of a short current-limiting stage at current densities ≤ 0.16 mA/cm^2^ (Figure 7). At the beginning of forward anodic polarization, a stage of active corrosion (stage 1), is succeeded by stage 2, a short current-limiting stage (~100 mV). Subsequently, there is a rapid current density rise, not as sharp as in low Co compositions, (stage 3), followed by stabilization at high values (stage 4). This polarization pattern in 3.5% NaCl has been noted in VAM-fabricated Al–Co alloys [19]. More analytically, the active stage was associated with preferential corrosion of the Al matrix, which is less noble than the Co aluminides. The subsequent current-limiting stage (stage 2) was associated with surface film formation on the Al matrix. The next current rise (stage 3) was mainly related to surface film breakdown at the Al/Al_9_Co_2_ interfaces leading to Al-matrix pitting. Current stabilizing at stage 4 resulted from the formation of unstable products, like hydrated Al oxides in pits and crevices, as well as the passivation of Al_9_Co_2_. 

Reverse scanning provides additional insights into the identified stages. Despite the clockwise hysteresis loop during reverse polarization through stage 3, confirming localized corrosion, the anodic portion’s gradients during this stage are not as flat as in the 0, 2, and 5 wt% Co cases (especially 0 and 2 wt% Co, see Figure 7b,c), indicating oxidation of Al_9_Co_2_, besides pitting, during this stage. The counter-clockwise hysteresis in the final stabilization stage, aligns with the temporary closure of the pits with hydrated Al oxides and Al_9_Co_2_ passivation. During reverse scanning through stage 3, the pits reopen, turning the counter-clockwise hysteresis to clockwise (Figure 7).

The clockwise hysteresis loop areas of the Al–10Co and Al–20Co voltammograms are smaller than those of Al–2Co and Al–5Co, while Al–32Co displays counter-clockwise hysteresis throughout the anodic scan. Furthermore, the E_a/c tr_ values are higher than E_corr_, indicating nobler surfaces at E_a/c tr_ compared to E_corr_. It should be noted that the nobility of E_a/c tr_ relative to the E_corr_ values indicates a relatively high resistance to localized corrosion based on the following considerations [35,36]: a nobler E_a/c tr_ compared to E_corr_ implies that the corroded surface turns to function as a cathode, and its dissolution is set back by the non-corroded surface; then, the non-corroded area preferentially corrodes. As a result, the corrosion of the alloy uniformly progresses throughout its surface. On the other hand, an E_a/c tr_ value less noble than E_corr_ indicates the continued anodic action of the corroded surface, leading to increased corrosion and, consequently, cathodic protection of the non-corroded surface. Therefore, the corrosion of the alloy advances to the depth inducing the formation of deep pits.

The smaller differences between the final cathodic currents of the reverse scan and the initial cathodic currents of the forward scan in high Co compositions also suggest their superior resistance to localized corrosion compared to low Co compositions. In Figure 7a and more clearly in Figure 7c, the reduced differences at the lowest potentials for the high Co alloys indicate less surface degradation after cyclic polarization (compare the cathodic current differences at the lowest potentials for Al and Al–10Co in Figure 7c).

It is thus concluded that Al– (10–32)Co alloys exhibit a relatively good resistance to localized corrosion forms, apparently associated with the increased Co content. 

**Electrochemical values:** The E_corr_ values align with the polarization behavior distinction between low Co compositions (2–5 wt% Co) and high Co compositions (10–32 wt% Co). The E_corr_ values for 2–5 wt% Co are very close to that of cast Al and are also close to their E_b_ value, corroborating the aforementioned observation that their polarization behavior is governed by that of the Al matrix. Conversely, the “10–32 wt% Co” alloys exhibit much lower E_corr_ values not because they are more reactive than the “2–5 wt% Co” alloys, but because the latter at their corrosion potential have already experienced pitting during the preceding cathodic polarization and/or during the initial open-circuit state [37,38]; consequently, at the onset of anodic polarization, the “2–5 wt% Co” alloys readily polarize at potentials close to their pitting potential, a common occurrence in aluminum alloys in saline environments [39,40]. As such, Al–32Co presents the lowest E_corr_ value as it has not suffered from pitting during the open-circuit state and/or cathodic polarization. This is also deduced from the counter-clockwise hysteresis loop of the anodic polarization curve.

Although the low resistance of the low Co compositions to localized corrosion makes the determination of i_corr_ (i.e., general corrosion rate) meaningless, the latter has been calculated for the sake of the following discussion. Table 2 shows that an increase in Co content correlates with a higher i_corr_, despite the corresponding increased noble IC phase content. This i_corr_ increase is attributed to the coarsening of the IC needles, since coarser cathodic surfaces can better support cathodic reactions. Furthermore, the increased ratio of “cathodic surface area-to-anodic surface area” intensifies corrosion cell activity. As corrosion progresses, this ratio increases even more as the anode is gradually consumed, further accelerating uniform corrosion [41,42].

Increasing Co content from 10 to 32 wt% Co raises the passive current density (stage 2), aligning with the corresponding decrease in the Al content and, hence, the passivated Al phase. As previously noted, the current-limiting stage 2 following the stage of Al active dissolution is owing to the passivation of the Al phase. 

**Figure 8:**
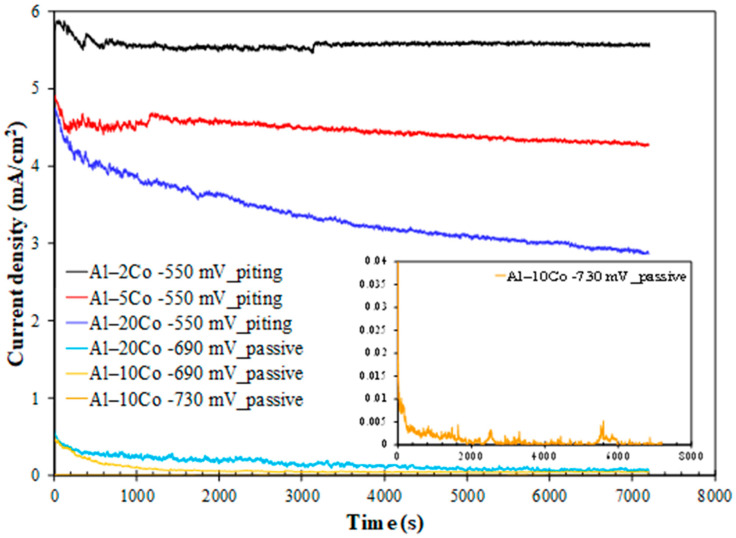
Chronoamperometry plots of Al–Co alloys at potentials in the passive-like and pitting stages (3.5 wt% NaCl (RT)). Inset: magnified plot for Al–10Co at −730 mV vs. Ag/AgCl.

**Chronoamperometry:** The current density against time curves during polarization at potentials in passive-like stage 2 and pitting stage 3 for the Al– (10–32)Co alloys and pitting stage b for the Al– (2-5) wt% Co alloys are illustrated in Figure 8. 

Polarization of Al–10Co and Al–20Co at potentials in current-limiting stage 2 manifests typical passive behavior: an initial sharp drop in the current density and, after reaching a minimum, relaxing at steady values. This pattern implies the growth of a surface film, reaching a maximum thickness and maintaining it thereafter. Stabilization at very low current density values confirms true passivity for 10–20 wt% Co, as suggested by the potentiodynamic curves in Figure 7. The anhydrous, amorphous oxide underlayer, situated between the metallic surface and the hydrated Al oxide top layer, is responsible for the very low current density values in passive-like stage 2 [18,19]. Hence, the shape of the “i vs. t” curves and the sequence of passive current values (Al–10Co < Al–20Co) corroborate the potentiodynamic polarization results (Figure 7 and Table 2), regarding the: (a) passivation during stage 2, and (b) sequence of passive currents. The higher current density fluctuation in the “i vs. t” curve of Al–20Co (−690 mV vs. Ag/AgCl) compared to Al–10Co (−690 mV vs. Ag/AgCl) is due to the closer polarization potential of Al–20Co to its E_b_ value, compared to Al–10Co, inducing more defective films.

Polarization at −550 mV vs. Ag/AgCl, a potential within steep current-increasing stages b (Al–2Co and Al–5Co) and 3 (Al-20Co) reveals the following trends: an instant initial current density rise is succeeded by a rapid drop (still at high values though), followed by stabilization (2 and 5 wt% Co), or a gradual, slow decrease (20 wt% Co). These trends along with the current density fluctuations in the test’s early stages (<1900 s), suggest that localized corrosion is associated with deposition of unstable products; accumulation of the latter (along with simultaneous passivation of the IC phase, as discussed next) suspends pitting as the potentiostatic test progresses. 

The positive impact of Co on Al–Co alloys’ localized corrosion resistance, manifested by the potentiodynamic tests, is also evidenced by the reduced current with increasing Co content during polarization at −550 mV vs. Ag/AgCl. At this potential, Al–20Co shows a sharper initial current density drop compared to Al–2Co and Al–5Co. Unlike Al–-5Co, with a sustained but insignificant drop, and Al–2Co, which exhibits no drop after initial stabilization, Al–20Co’s current density continues to decline throughout the test. These trends can be justified by the simultaneous oxidation/passivation of the IC phase, the extent of which (i.e., the extent of the IC phase) follows the order: Al–20Co >> Al–5Co > Al–2Co.

### 3.4. Electrochemical Behavior of the Heat-Treated Alloys

**Figure 9:**
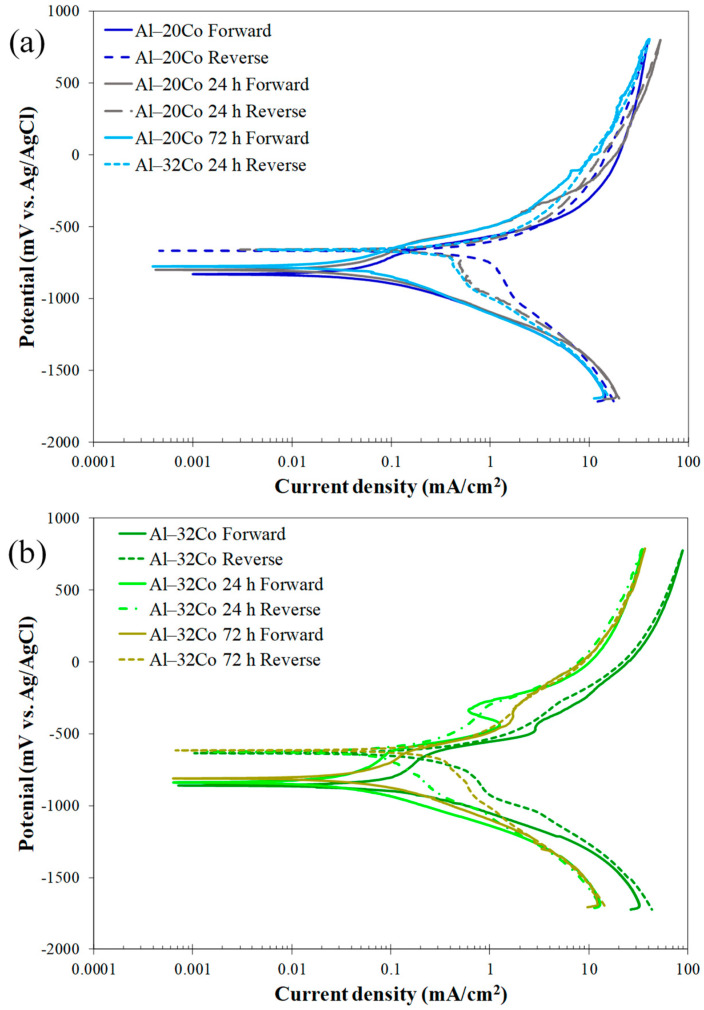
Potentiodynamic polarization curves in 3.5 wt% NaCl (RT) of: (**a**) Al–20Co, (**b**) Al–32Co, as cast and after heat treatment at 600 °C.

Figure 9 displays the potentiodynamic polarization curves of Al–20Co (Figure 9a) and Al–32Co (Figure 9b) in both as-cast and heat-treated conditions. While the similar shapes of the curves indicate similar corrosion mechanisms, the anodic polarization portions of the HT alloys shift to slightly lower current density values at higher potentials. Nonetheless, the differences are small, as shown by the similar electrochemical values, especially for Al–20Co (Table 3). The only accountable and consistent difference is a modest increase in the E_corr_ values with the HT time, reflecting stress relief during annealing. Stresses, whether from external loads or residual stresses, can raise the internal energy, leading to a decrease in the electrochemical potential [43]. The corrosion current density has modestly decreased with the HT due to stress relief and particle coarsening/rounding due to sintering. The increase in the i_corr_ value of Al–32Co after 72 h of HT is mainly attributed to the relatively high porosity caused by the Kirkendall effect.

The impact of the Al_13_Co_4_ to Al_9_Co_2_ transformation on the corrosion rate cannot be determined with confidence. Galvanic effects between Al_13_Co_4_ and Al are absent due to the lack of electrical/physical contact. The galvanic effect between Al_13_Co_4_ and Al_9_Co_2_ has been proven to be insignificant [18]. On the other hand, the elimination of Al_13_Co_4_ blades may be linked to stress relaxation, leading to reduced corrosion current density.

### 3.5. Microstructure of Corrosion

**Figure 10:**
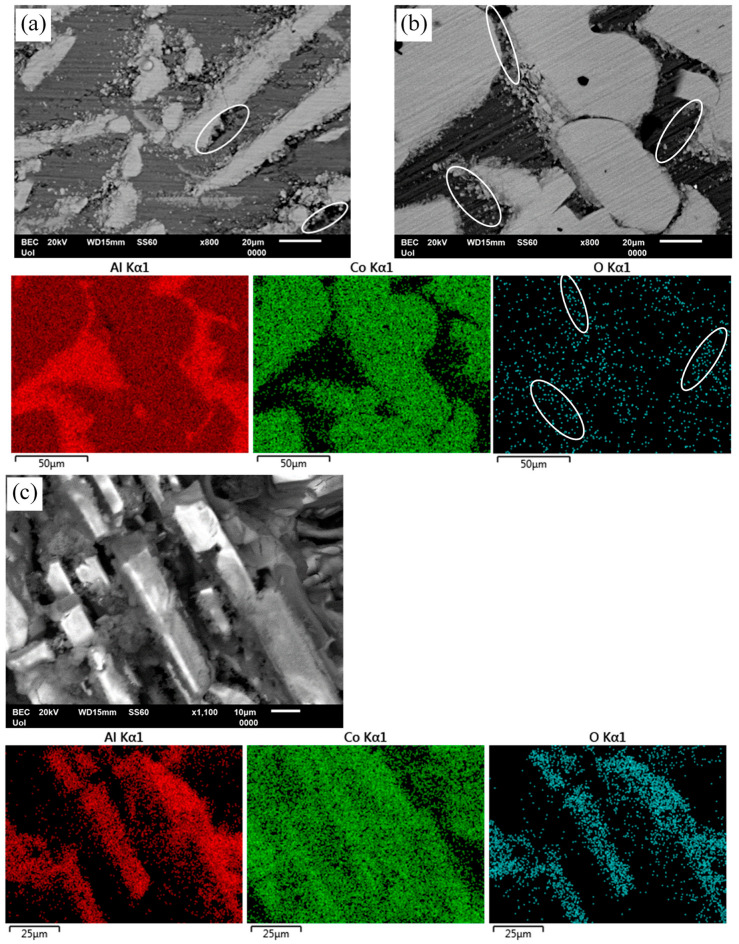
Surface states of Al-Co alloys after chronoamperometry (**a**,**b**) and cyclic polarization (**c**) in 3.5 wt% NaCl (RT). (**a**) Al–5Co (−660 mV vs. Ag/AgCl-active stage 1), (**b**) Al–20Co (−690 mV vs. Ag/AgCl current-limiting stage 2); (**c**) Al–10Co. Ellipses in (**a**) point at Al pitting at Al_9_Co_2_/Al boundaries. Ellipses in (**b**) and its oxygen map denote passivation of the Al-matrix, especially near Al_9_Co_2_/Al boundaries. Preferential dissolution of the Al-matrix and passivation of the intermetallic blades are shown in (**c**).

Figure 10a presents the surface state of Al–5Co after chronoamperometry in 3.5 wt% NaCl, at −660 mV vs. Ag/AgCl, a potential in active stage 1. The presence of pits (some outlined in white ellipses) confirms the suggestion in Section 3.3 (“Electrochemical values” subsection) that, especially for the “2–5 wt% Co” alloys, Al pitting has already occurred during the preceding cathodic polarization, or even during the open-circuit state. 

Figure 10b illustrates the surface state of Al–20Co and the EDX elemental maps after chronoamperometry in 3.5 wt% NaCl (RT), at −690 mV vs. Ag/AgCl, a potential in current-limiting stage 2. Passivation of the Al matrix, especially at the Al_9_Co_2_/Al boundaries (see ellipses in the electron micrograph and the oxygen map), suggests that dissolution of Al has started at the interfaces due to the electrochemical potential difference between Al and Al_9_Co_2_. (The latter, as both an intermetallic compound and a complex metallic alloy, is quite nobler than Al metal.) Passivation of Al follows. The Al_9_Co_2_ surface has remained oxidation free, confirming the suggestion in Section 3.3 (“High-Co compositions” subsection) that the first current-limiting stage is owing to the passivation of the Al matrix, given the notably more negative free energies of formation of aluminum oxides, hydroxides, and oxyhydroxides compared to the free energies of formation of cobalt oxides, hydroxides, and oxyhydroxides [19].

Figure 10c shows the surface state of Al–10 wt% Co and the EDX elemental maps after cyclic polarization in 3.5 wt% NaCl. The protrusion of Al_9_Co_2_ blades manifests the preferential dissolution of the Al matrix. Oxidation of the Al_9_Co_2_ blades implies passivation of Al_9_Co_2_ at the final current-limiting stage (stage 4), as postulated in Section 3.3 (“High-Co compositions” subsection). The preferential dissolution of Al is owing to the large electromotive force difference between Al and intermetallic Al_9_Co_2_, and the dissolution tendency of Al_2_O_3_ at alkaline pHs [44] (Local alkalinity is risen due to OH^−^ generation by oxygen reduction on Al_9_Co_2_ at the Al_9_Co_2_/Al boundaries). 

**Figure 11:**
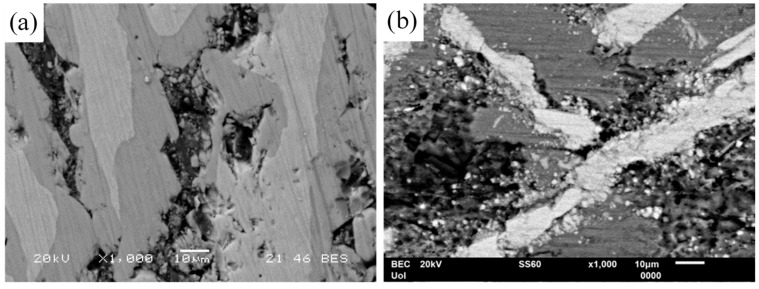
Surface state of (**a**) Al–32Co and (**b**) Al–5Co after cyclic polarization in 3.5 wt% NaCl (RT), displaying selective dissolution of the Al-matrix. In Al–32Co, crevices are filled with intermetallic fragments.

**Figure 12:**
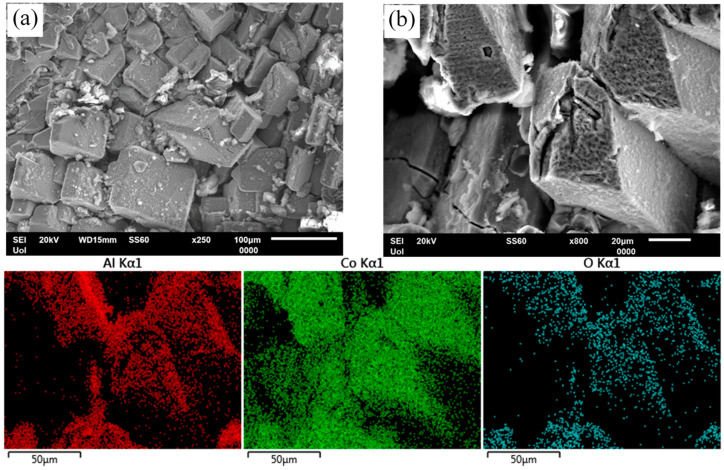
Surface state of Al–20Co, heat treated at 600 °C, after cyclic polarization in 3.5 wt% NaCl (RT). (**a**) Al–20Co (72 h), (**b**) Al–20Co (24 h), and EDX elemental maps. In both cases, preferential dissolution of the Al matrix and oxidation of the IC particles are observed.

Based on the electrochemical findings, microstructure examination, and prior research [6,41], a corrosion mechanism is proposed: The elevated alkalinity at the Al_9_Co_2_/Al interface causes dissolution of the Al_2_O_3_ film on Al which is further degraded by chlorine adsorption. Galvanic cells form between the exposed metal and adjacent IC initiating small pits where agile chlorine ions induce hydrolysis reactions. The resulting acidification of the pit environment leads to further pit growth, as Al_2_O_3_ is also soluble in acidic environments [44]. Pit growth and coalescence ultimately result in crevice corrosion, discerned in Figure 10c, Figure 11a and Figure 12a.

The passivation of Al_9_Co_2_ constitutes a major reason for the superior localized resistance of high-Co alloys compared to low-Co alloys, evident in the chronoamperometry plots of Figure 8 at pitting stage 3. The film on Al_9_Co_2_ comprises amorphous Co-oxide and Al-hydroxides/oxyhydroxides [18]. The beneficial impact of this mixed, multilayer structure of passive oxides on aluminides of Ni and Fe (adjacent elements to Co in the same periodic table period), where the inner layer acts a barrier, has been previously acknowledged [45,46]. The incorporation of transition metals, like Fe [46], Mo [47], W [48], Cr and Ti [49] in the passive film of Al, has demonstrated enhanced passivity in acidic [46,47] and chloride-containing electrolytes [47,48,49].

Figure 11 highlights another reason for the superior resistance of high Co alloys to localized corrosion compared to low Co alloys. The higher the Co content, the coarser the brittle IC blades and the narrower the supporting Al zones. The latter have embrittled due to oxidation. The coarser a brittle particle, the more susceptible to cracking when a critical particle size is exceeded [50]. Surface micrographs of Al-32Co (Figure 11a) and Al-5Co (Figure 11b) after cyclic polarization reveal selective dissolution of the Al-matrix. In Al-32Co, selective dissolution and embrittlement of the Al-matrix weakens the support to the IC blades causing peripheral fragmentation. The gaps generated from Al dissolution, fill with IC fragments (Figure 11a) obstructing the electrolyte route and outward Al^3+^ diffusion. Conversely, in Al-5Co, the high Al-to-Al_9_Co_2_ surface area ratio prevents IC fragments from filling large pits in the Al-matrix (Figure 11b). 

Figure 12 illustrates the surface state of HT alloys after cyclic polarization. In Figure 12a, Al-20Co (600 °C, 72 h), shows selective dissolution of the Al-matrix. At higher magnification, Figure 12b (HT Al-20Co, 600 °C, 24 h) and the respective EDX elemental maps reveal that the Al_9_Co_2_ particles have been oxidized, probably in current-increasing stage 3 and, then, passivated in current-limiting stage 4. 

To conclude, the microstructural observations corroborate the results of both potentiodynamic and potentiostatic testing, as well as the corrosion stages claimed in Section 3.3 (“High Co compositions” subsection), confirming the similarity of the corrosion processes in both as-cast and heat-treated alloys.

## 4. Conclusions

The following conclusions are drawn from investigating Al–Co alloys (2–32 wt% Co) fabricated by stir casting with KBF_4_ flux:

- Al– (2-10) wt% Co alloys are composed of acicular Al_9_Co_2_ particles uniformly dispersed in an Al matrix. The size of the aluminides ranges from fine needles to coarse wedges with increasing Co content. Al–20Co and Al–32Co alloys additionally feature Al_13_Co_4_ blades within Al_9_Co_2_ wedges. Al–32Co presents an almost entirely intermetallic structure composed of Al_9_Co_2_ wedges engulfing Al_13_Co_4_ lumps and thin Al stringers “anchoring” the Al_9_Co_2_ wedges.
- Heat treatment of Al–20Co and Al–32Co at 600 °C for up to 72 h significantly reduces the volume fraction of Al_13_Co_4_ through transformation to Al_9_Co_2_, aided by annealing and Al diffusion through porosity from sintering. Simultaneously, Al reacts with free Co, increasing the volume fraction of Al_9_Co_2_. The hardness of the alloys decreases with the heat-treatment time, more prominently in Al–32Co.
- Based on their polarization performance in 3.5 wt% NaCl, the studied alloys are categorized as low Co (Al–2Co and Al–5Co) and high Co (Al– (10–32)Co). High Co compositions exhibit enhanced resistance to localized corrosion, attributed to the increased surface area of Co-stabilized surface films on aluminides and the accumulation of intermetallic fragments in the gaps generated from Al dissolution. Conversely, the resistance to uniform corrosion decreases with increasing Co content, attributed to coarser cathodic intermetallic particles.
- Heat-treated alloys present electrochemical behavior similar to their as-cast counterparts. Noteworthy effects of heat treatment on Al–20Co and Al–32Co include increased corrosion potential values, reduced corrosion rates, and shifts of anodic polarization curves to lower current values. These beneficial effects stem from stress-cell reduction through annealing and Al_9_Co_2_ wedge rounding due to sintering/annealing.
- Combining the potentiodynamic and potentiostatic behavior with microstructural observations, a corrosion mechanism is proposed for high Co alloys (Al– (10–32)Co)), as cast and heat treated, involving the following stages: (i) active corrosion by Al-phase dissolution; (ii) passivation of the Al phase; (iii) breakdown of the passive film at the Al/Al_9_Co_2_ interfaces, pitting of Al, and oxidation of Al_9_Co_2_; (iv) passivation of Al_9_Co_2_ and formation of unstable films on the Al phase. For low Co alloys (Al–2Co and Al–5Co), stages (i)–(iii) have already occurred, to an extent, during the preceding cathodic polarization and/or open-circuit state.

## Figures and Tables

**Table 1:** Hardness values (HB2.5) of Al–Co alloys in the as-cast and heat-treated states.

Alloy	As Cast	600 °C 24 h	600 °C 48 h	600 °C 72 h
Al–2Co	41 ± 2	38 ± 1	37 ± 1	-
Al–5Co	47 ± 2	48 ± 2	44 ± 3	-
Al–10Co	55 ± 4	-	52 ± 5	-
Al–20Co	95 ± 8	86 ± 11	84 ± 10	80 ± 5
Al–32Co	188 ± 19	115 ± 31	97 ± 19	88 ± 13

**Table 2:** Electrochemical values of the alloys in 3.5 wt% NaCl (RT). E_corr_, E_cp_, E_b_, E_a/c tr_: corrosion, critical passivation, breakdown, anodic-to-cathodic transition potential, respectively; i_corr_, i_p_: corrosion current density, current density in the middle of current-limiting stage 2.

Alloy	E_corr_ (mV vs. Ag/AgCl)	E_cp_ (mV vs. Ag/AgCl)	E_b_ (mV vs. Ag/AgCl)	E_a/c tr_ (mV vs. Ag/AgCl)	i_corr_ (mA/cm^2^)	i_p_ (mA/cm^2^)
Al1050	−703	-	−656	−720	-	-
Al–2Co	−673	-	−610	−674	0.013	-
Al–5Co	−686	-	−618 *	−657	0.034	-
Al–10Co	−801	−756	−658	−653	0.062	0.06
Al–20Co	−833	−776	−672	−671	0.075	0.09
Al–32Co	−860	−750	−650	−625	0.130	0.16

* Eb_1_ = −618 mV vs. Ag/AgCl; Eb_2_: −578 mV vs. Ag/AgCl.

**Table 3:** Electrochemical values of the alloys immersed in 3.5 wt% NaCl (RT). E_corr_: corrosion potential, E_cp_: critical passivation potential, E_b_: breakdown potential, E_a/c tr_: anodic-to-cathodic transition potential, i_corr_: corrosion current density, i_p_: current density in the middle of current-limiting stage 2.

Alloy	E_corr_ (mV vs. Ag/AgCl)	E_cp_ (mV vs. Ag/AgCl)	E_b_ (mV vs. Ag/AgCl)	E_a/c tr_ (mV vs. Ag/AgCl)	i_corr_ (mA/cm^2^)	i_p_ (mA/cm^2^)
Al–20Co	−833	−776	−672	−671	0.075	0.10
Al–20Co 24 h	−801	−740	−645	−662	0.063	0.09
Al–20Co 72 h	−777	−730	−633	−663	0.066	0.08
Al–32Co	−860	−750	−650	−625	0.130	0.16
Al–32Co 24 h	−839	−740	−630	−626	0.035	0.08
Al–32Co 72 h	−811	−721	−641	−616	0.074	0.10

## Data Availability

Data sharing is not applicable to this article.
